# Characteristic of molecular subtypes based on PANoptosis-related genes and experimental verification of hepatocellular carcinoma

**DOI:** 10.18632/aging.204720

**Published:** 2023-05-12

**Authors:** Haitao Ren, Na Kang, Shuan Yin, Chen Xu, Tengfei Qu, Dongdong Dai

**Affiliations:** 1 Department of Interventional Medicine, The Affiliated Hospital of Qingdao University, Qingdao, Shangdong 266071, China; 2 Operating Room, The Affiliated Hospital of Qingdao University, Qingdao, Shangdong 266071, China; 3 Department of Pediatrics, The Affiliated Hospital of Qingdao University, Qingdao, Shangdong 266071, China; 4 Department of Infectious Disease, The Affiliated Hospital of Qingdao University, Qingdao, Shangdong 266071, China; 5 Department of Hepatobiliary and Pancreatic Surgery, The Affiliated Hospital of Qingdao University, Qingdao, Shangdong 266071, China

**Keywords:** hepatocellular carcinoma, PANoptosis-related genes, risk model, immune infiltration, molecular subtypes

## Abstract

Hepatocellular carcinoma (HCC) is a type of liver cancer that originates from liver cells. It is one of the most common types of liver cancer and a leading cause of cancer-related death worldwide. Early detection and treatment can improve the HCC prognosis. Therefore, it is necessary to further improve HCC markers and risk stratification. PANoptosome is a cytoplasmic polymer protein complex that regulates a proinflammatory programmed cell death pathway called “PANoptosis”. The role of PANoptosis in HCC remains unclear. In this study, the molecular changes of PANoptosis related genes (PAN-RGs) in HCC were systematically evaluated. We characterized the heterogeneity of HCC by using consensus clustering to identify two distinct subtypes. The two subtypes showed different survival rate, biological function, chemotherapy drug sensitivity and immune microenvironment. After identification of PAN-RG differential expression genes (DEGs), a prognostic model was established by Cox regression analysis using minimum absolute contraction and selection operator (LASSO), and its prognostic value was verified by Cox regression analysis, Kaplan-Meier curve and receiver operating characteristic (ROC) curve. Our own specimens were also used to further validate the prognostic significance and possible clinical value of the selected targets. Subsequently, we conducted a preliminary discussion on the reasons for the influence of the model on the prognosis through TME analysis, drug resistance analysis, TMB analysis and other studies. This study provides a new idea for individualized and precise treatment of HCC.

## INTRODUCTION

Hepatocellular carcinoma (HCC) is a type of liver cancer that originates from hepatocytes. It is one of the most common types of liver cancer and a leading cause of cancer-related deaths worldwide [1]. Risk factors for HCC include chronic liver disease, such as cirrhosis, hepatitis B and C, and excessive alcohol consumption [2]. Treatment options include surgery, radiation therapy, chemotherapy, and targeted therapy [1, 3]. Early detection and treatment can improve outcomes due to poor therapeutic outcomes in advanced HCC [4].

HCC prognosis is influenced by a variety of factors, including the tumor microenvironment (TME), which interacts with the cancer cells and can affect the growth and spread of the tumor [5]. TME that can impact HCC prognosis by multiple ways, including inflammation, angiogenesis, immune response stromal cells and extracellular matrix. Chronic inflammation can contribute to the development and progression of HCC [6]. The formation of new blood vessels in the TME can provide the tumor with nutrients and oxygen needed for growth. The immune system’s response to the tumor can impact the prognosis [7]. A strong immune response can slow tumor growth, while a weak immune response can allow the tumor to grow unchecked [8]. The stromal cells in the TME, such as fibroblasts and myofibroblasts, can influence tumor growth and progression [9]. The extracellular matrix, the network of proteins and carbohydrates that surrounds the cells, can influence the behavior of cancer cells and impact the prognosis [10]. Due to the current limited research status, analysis of TME can further explore the risk factors associated with HCC prognosis, with a view to better risk stratification of HCC patients.

PANoptosome is a cytoplasmic polymer protein complex containing receptor interacting protein kinase (RIPK1), ASC, and caspase-8 [11]. The pro-inflammatory programmed cell death pathway regulated by PANoptosome is called “PANoptosis” [12], highlighting the crosstalk and coordination that occurs between pyroptosis, apoptosis and necroptosis [13, 14]. PANoptosis plays a very complex role in tumor progression, cancer therapy and cancer immune regulation [15]. Patterns of PANoptosis can predict immunotherapy response and patients’ survival in multiple cancer types, including gastric cancer, colon cancer and lung cancer [16–18]. One possible mechanism is that the effect of IFN treatment can be affected by PANoptosis [19]. There is evidence shown that complexes formed by AIM2, caspase-8, ZBP1, RIPK3, RIPK1 and FADD can sense PAMP, DAMP or other risk factors and drive PANoptosis [20]. Among these PANoptosis driver proteins, AIM2 has been reported to play an inhibitory role in regulating the growth and metastasis of HCC by regulating immune cell infiltration, indicating that AIM2 can be used as a potential therapeutic target for HCC [21, 22]. As another PANoptosis driver protein, ZBP1 has been shown to be expressed in interferon (IFN)-treated HCC, which indicated its potential HCC-related function [23]. The involvement of other PANoptosis driver protein, including NLRP3, RIPK3, RIPK1 and FADD, was also reported in HCC [24–27]. Although most PANoptosis driver proteins have been unveiled the role in HCC, the impact of PANoptosis on HCC carcinogenesis remains unknown. It is of great significance to further study the role of PANoptosis in HCC and clarify its carcinogenic or anticancer effect.

In this study, seven PANoptosis-related genes (PAN-RGs) were selected, and the prognostic model of liver cancer was established successfully. Significance of prognostic prediction and the possible clinical value of selected targets were further verified through public databases and our own clinical specimens. It provides a new idea for individualized and precise treatment of HCC.

## MATERIALS AND METHODS

### Data acquisition

Two transcriptome cohorts of HCC samples were acquired from the public databases. The TCGA-LIHC cohort was downloaded from the TCGA database (https://portal.gdc.cancer.gov/), including 424 samples (50 normal and 374 HCC samples). The GSE76427 contains 115 HCC samples was acquired from the GEO database. In first, the matrix format of TCGA-LIHC was transformed from the FKPM into TPM, and the two transcriptome expression profiles were merger into a final file via package “sva” [28]. The matching clinical information of HCC was obtained from the TCGA and GEO database and the HCC samples without survival time were deleted in this study, and 485 HCC samples were included in total [29].

### Analysis of copy number variation and somatic mutation

The PANoptosis-related genes (PAN-RGs) were acquired from the previous literature, and 14 PAN-RGs were collected (Supplementary Table 1). The expression of PAN-RGs in normal and HCC samples were extracted via “limma” package, and the threshold for differential analysis was set at |fold change| > 1, *p* < 0.05. The copy number variation (CNV) data and somatic mutation files (maf format) of HCC samples were downloaded from the TCGA database. R package “circos” was utilized to explore the location of PAN-RGs on chromosome based on the gene reference file. The potential protein-protein interaction (PPI) of PAN-RGs was explored using STRING database.

### Exploration of PAN-RG-based molecular subgroups via consensus cluster algorithm

Firstly, we extracted the expression profile of PAN-RGs from the merge file (TCGA-LIHC and GSE76427). “ConsensusClusterPlus” R package was developed to cluster the HCC samples into different molecular subgroups with the max K set at 9. Under the best classification, the HCC samples were divided into PAN-RG cluster A, B and C. R script “survival” was conducted to display the clinical prognosis for HCC samples in different unsupervised subgroups. Principal component analysis (PCA) was used to explore the intergroup difference between the different PAN-RG cluster subgroups via “ggplot2”. Pheatmap script was adopted to exhibit the relationship of PAN-RGs expression profile and clinical variates for HCC samples.

### Evaluation of immune infiltration landscape and immunotherapy response

We developed single sample gene set enrichment analysis (ssGSEA) to evaluate the immune infiltration of each HCC sample. On the basis of 23 immune cells gene marker, the proportion of 23 immune cells was estimated via script “GSVA”. In addition, the immune status was investigated using “estimate” script in R environment. The IPS file included PD-1 and CTLA-4 treatment data was downloaded from The Cancer Immunome Atlas (TCIA) database.

### Analysis of functional enrichment for PAN-RG cluster-based DEGs

Based on the selection threshold of |fold change | > 1, *p* < 0.001, the intersection differential expression genes (DEGs) between the PAN-RG subgroups were acquired. “clusterProfiler” R script was developed to enrich the PAN-RG cluster-based DEGs into different molecular biological (GO and KEGG term) (*p* < 0.05) [30]. Refer to the gene list of different KEGG terms, the KEGG term of HCC samples in PAN-RG cluster subgroups was calculated using “GSVA” algorithm.

### Generation of risk subgroups based on the prognostic DEGs

In first, the expression profile of the intersection DEGs was obtained and conducted a univariate Cox analysis. Then, based on the expression of prognostic DEGs, “ConsensusClusterPlus” script was employed to distinct the HCC samples into different gene-cluster subgroups. According to the multivariate Cox analysis and “caret” script, the independent prognostic variates were obtained and divided the HCC samples into training cohort and test cohort under the division cutoff set at 1:1 [31]. In accordance with the expression profile and coefficient of the independent prognostic variates, the risk score of each HCC sample was calculated.

### Evaluation of independent prognosis and establishment of nomogram

The clinical features of HCC samples were enrolled from the TCGA-LIHC and GSE76427. To explore the independence of the risk score for HCC, we conducted a univariate and multivariate Cox analysis in the entire, training and test cohorts. Receiver operating characteristic curve (ROC) was used to explore the AUC of risk score, age, gender and HCC-based stage, respectively. Based on those clinical features and risk score, the nomogram model was developed to evaluate the 1-, 3-, and 5-years clinical survival outcome for HCC samples. Decision curve analysis (DCA) was used to appraise the accuracy of nomogram and other indicators in predicting clinical prognosis via “ggDCA” script.

### Analysis of mutation landscape and chemotherapeutic drug identification

MAF files of somatic mutation for HCC were downloaded from the TCGA database. Perl script was utilized to extract the mutation data of HCC samples from the MAF files. “maftools” script was employed to display the somatic mutation frequency in risk subgroups for HCC. The response to chemotherapeutic drug for HCC samples was estimated using GDSC database via script pRRophetic.

### Real-time quantitative fluorescence PCR (qRT-PCR)

The experiment was approved by the Human Ethics Committee of the Affiliated Hospital of Qingdao University and the Ethics Office of Qingdao University. Tumor tissues and paired adjacent tissues were taken from HCC patients. Trizol reagent (Cat# 15596018, Thermo) was used to extract RNA from tumor tissues and adjacent tissues. The cDNA was synthesized using RT kit with gDNA Eraser (Perfect Real Time), and real-time quantitative qRT-PCR (Cat# RR047A, Takara) was performed. mRNA expression was detected by SYBR Pre-mix Ex Taq II (TliRNaseH Plus) (Cat# RR820B, Takara). Gene specific primer pairs are listed in Supplementary Table 2.

### Statistical analysis

Under the R language environment (R software 4.1.0), the data processing and difference analysis were carried out. Wilcoxon test was employed to calculate the statistical difference in two groups. Between multiple groups, ANOVA was utilized for statistical analysis. Spearman correlation analysis was performed to imply the relationship between the different variates. *P* < 0.05 was considered as statistical different in this study.

### Data availability statement

All data and clinical information involved in this paper were obtained from a public database, approved from the Ethics committee and written informed consent from patients were not required.

## RESULTS

**Figure 1:**
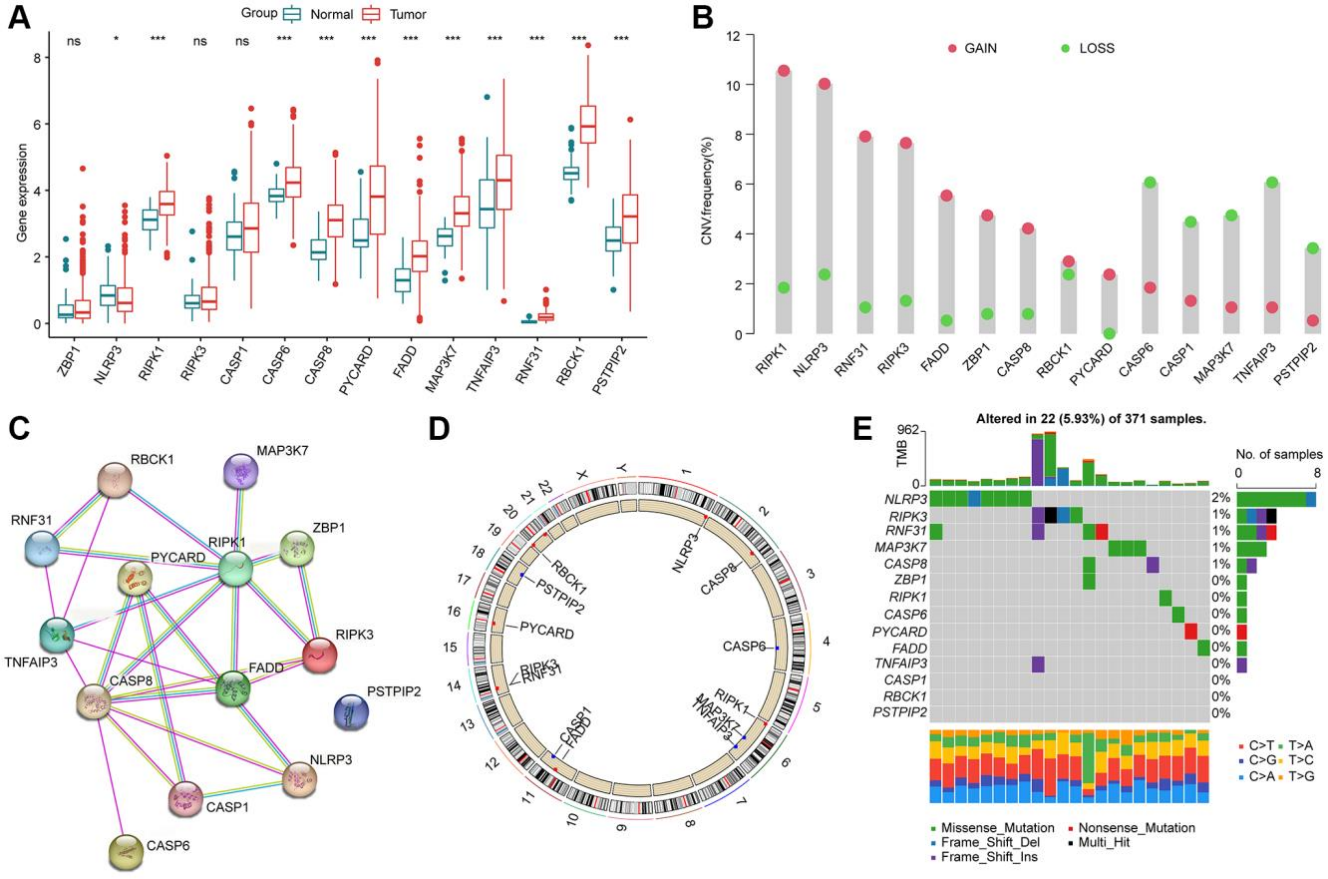
**Genetic expression and variation landscape of PAN-RGs in HCC.** (**A**) Genetic expression of 14 PAN-RGs in normal and HCC samples. (**B**) CNV exploration of PAN-RGs. (**C**) Interaction network of 14 PAN-RGs. (**D**) The location of PAN-RGs on chromosome. (**E**) Mutation evaluation of PAN-RGs in HCC samples.

To determine the character of PAN-RGs in the tumor progression of HCC, we acquired 14 PAN-RGs expression profile from the TCGA database. After the calculation of differential analysis, we observed that RIPK1, CASP6, CASP8, PYCARD, FADD, MAP3K7, TNFAIP3, RNF31, RBCK1, and PSTPIP2 were overexpressed in the HCC samples, nevertheless, the expression of NLRP3 was down expressed in the HCC samples (Figure 1A). On the basis of CNV file from the TCGA database, the CNV landscape of 14 PAN-RGs were explored and the result suggested higher amplification of RIPK1, NLRP3, RNF31, RIPK3, FADD, ZBP1, CASP8, and PYCARD, whereas CASP6, CASP1, MAP3K7, TNFAIP3, and PSTPIP2 showed higher deletion (Figure 1B). The PPI network illustrated a clear interaction of the 14 PAN-RGs (Figure 1C). In addition, the circle diagram displayed the location of the PAN-RGs on chromosome (Figure 1D). Mutation feature of PAN-RGs exhibited that the mutation frequency of NLRP3, CASP8, MAP3K7, RNF31 and RIPK3 was 2%, 1%, 1%, 1%, and 1%, respectively (Figure 1E).

### Molecular subtypes characteristic of HCC based on the PAN-RGs

**Figure 2:**
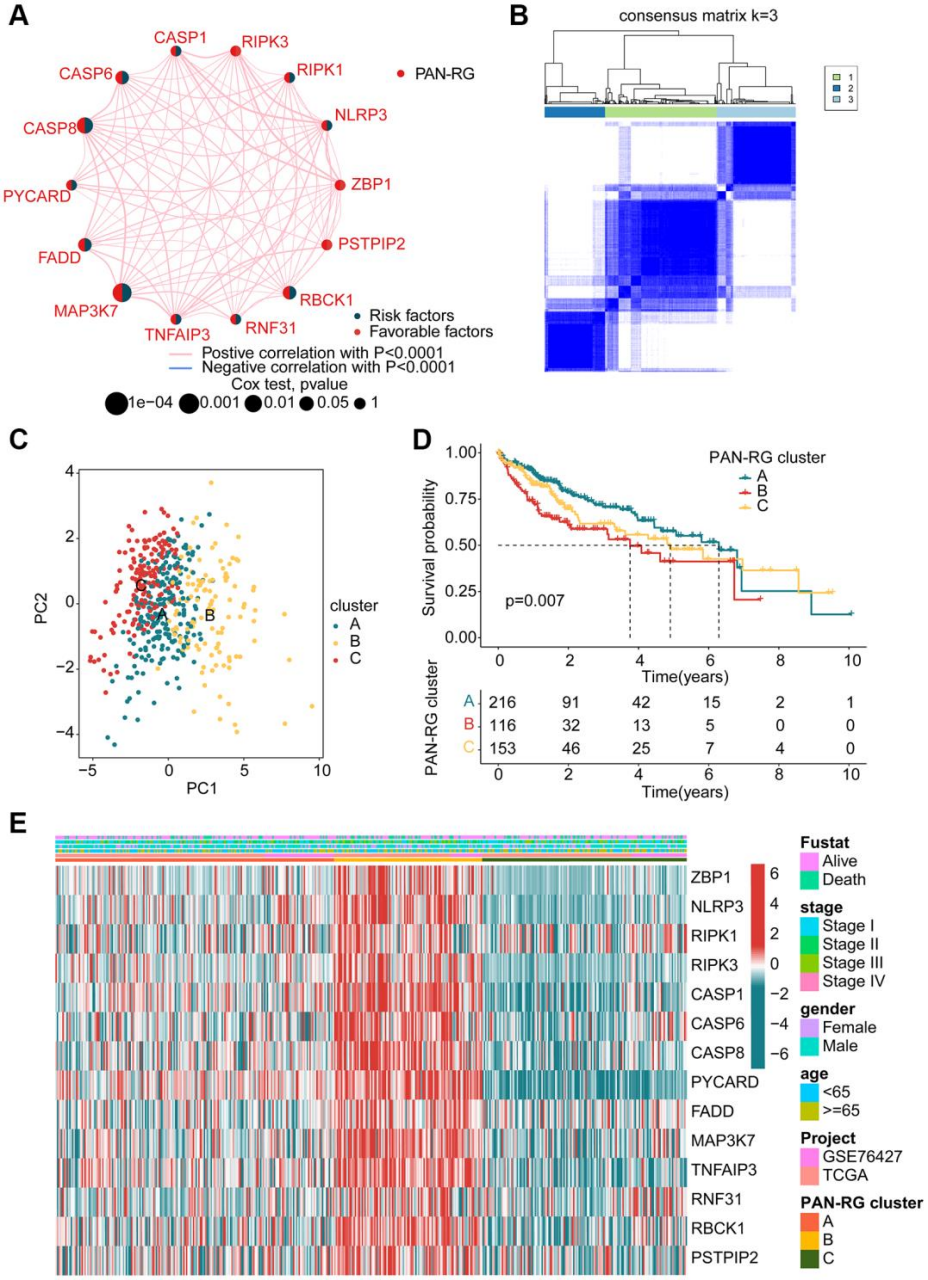
**Identification of molecular subtypes of PAN-RGs for HCC.** (**A**) Prognostic factor exploration of PAN-RGs. (**B**) Unsupervised consensus clustering analysis. (**C**) PCA diagram of HCC samples in cluster A, B, and C. (**D**) Clinical prognostic outcome of HCC in different PAN-RG cluster subgroups. (**E**) Expression of PAN-RGs in different PAN-RG cluster subgroups and clinical features.

In order to explore the molecular subtypes character of HCC, we enrolled 485 HCC samples to explain the relationship of PAN-RGs and tumorigenesis from the TCGA-LIHC dataset and GSE76427. The network plot illustrated the association of PAN-RGs and prognostic value. As shown in Figure 2A, a positive correlation was observed between the 14 PAN-RGs, and 5 prognostic risk factors were obtained (MAP3K7, CASP8, RBCK1, FADD, CASP6). After the estimated of an unsupervised consensus clustering analysis, the HCC samples displayed an optimal categorization of k = 3, with 216 HCC samples in PAN-RG cluster A, 116 HCC samples in PAN-RG cluster B, and 153 HCC samples in PAN-RG cluster C (Figure 2B). The PCA diagram displayed that the HCC samples in the different PAN-RG clusters could be clearly distinguished, indicating the accuracy of consensus clustering analysis (Figure 2C). Between the molecular subtypes of HCC, we observed a clear difference of prognosis in the different cluster subgroups, which the clinical prognosis of HCC in the cluster A was better than in cluster B and C (Figure 2D). The association of PAN-RGs and clinical features was displayed in a heatmap diagram, and the plot illustrated that the expression of PAN-RGs was greatly higher in PAN-RG cluster B (Figure 2E). These results demonstrate that the HCC samples could be accurately classify into different molecular subtypes based on the PAN-RGs expression, and associated with clinical prognosis for HCC.

### Exploration of immune infiltration in PAN-RG molecular subtypes

**Figure 3:**
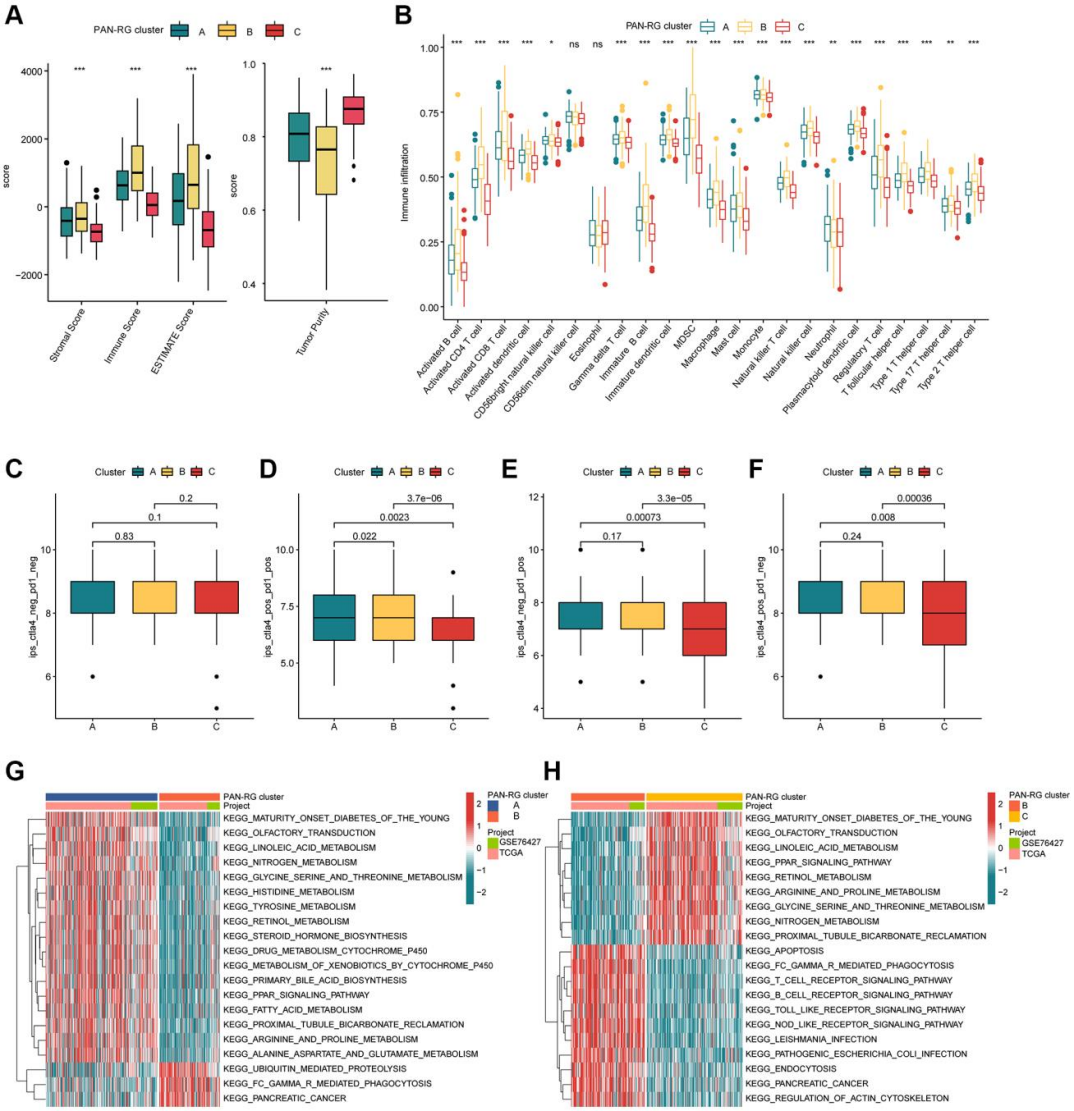
**The TME characteristic of HCC in the PAN-RG molecular subgroups.** (**A**) Evaluation of ESTIMATE score in PAN-RG cluster subtypes. (**B**) The immune infiltration exploration of HCC in PAN-RG cluster A, B, and C. (**C**–**F**) Immunotherapy response investigation of PAN-RG molecular subgroups via TCIA database. (**G**, **H**) GSVA algorithm shows the different regulation KEGG pathways in PAN-RG cluster A, B, and C.

The TME feature of HCC samples in the different PAN-RG molecular subtypes was explored in the next study. On the basis of ESTIMATE algorithm, the proportion of stromal and immune cells in different PAN-RG cluster subgroups was evaluated. The ESTIMATE result revealed that the HCC samples in PAN-RG cluster B had higher stromal, immune, ESTIMATE scores, and lower tumor purity (Figure 3A). A remarkable distinction was explored in the immune infiltration of 23 kind immune cells in PAN-RG cluster A, B, and C via ssGSEA assessment (Figure 3B). Furthermore, giving the significant difference in immune infiltration, we further estimated the immunotherapy response of HCC in PAN-RG cluster A, B, and C. The IPS assessment analysis revealed that the HCC samples in the PAN-RG cluster C displayed a worse immunotherapy response to PD-1 and CTLA-4 than PAN-RG cluster A and B (Figure 3C–3F). According to the GSVA algorithm, the regulatory role of crucial KEGG in the development of HCC was explored. Between PAN-RG cluster A and B, several metabolism-associated pathways were greatly down-regulated of HCC in the PAN-RG cluster B, involving in linoleic acid metabolism, histidine metabolism, tyrosine metabolism, retinol metabolism, and fatty acid metabolism. Of note, some tumor-associated signaling pathways were up-regulated for those poor prognosis HCC samples in PAN-RG cluster B, such as ubiquitin mediated proteolysis, Fc gamma R-mediated phagocytosis and pancreatic cancer (Figure 3G). In PAN-RG cluster C, a series of down-regulation immune-associated pathways were explored, such as T cell receptor signaling pathway, B cell receptor signaling pathway, Toll like receptor signaling pathway, NOD like receptor signaling pathway (Figure 3H). These discoveries exhibit that the PAN-RG-based molecular subtypes of PAN-RG were closely associated with immune infiltration, and could reveal the immunotherapy response of HCC in different PAN-RG molecular subgroups.

### Generation of DEGs and gene-cluster subgroups identification for HCC

**Figure 4:**
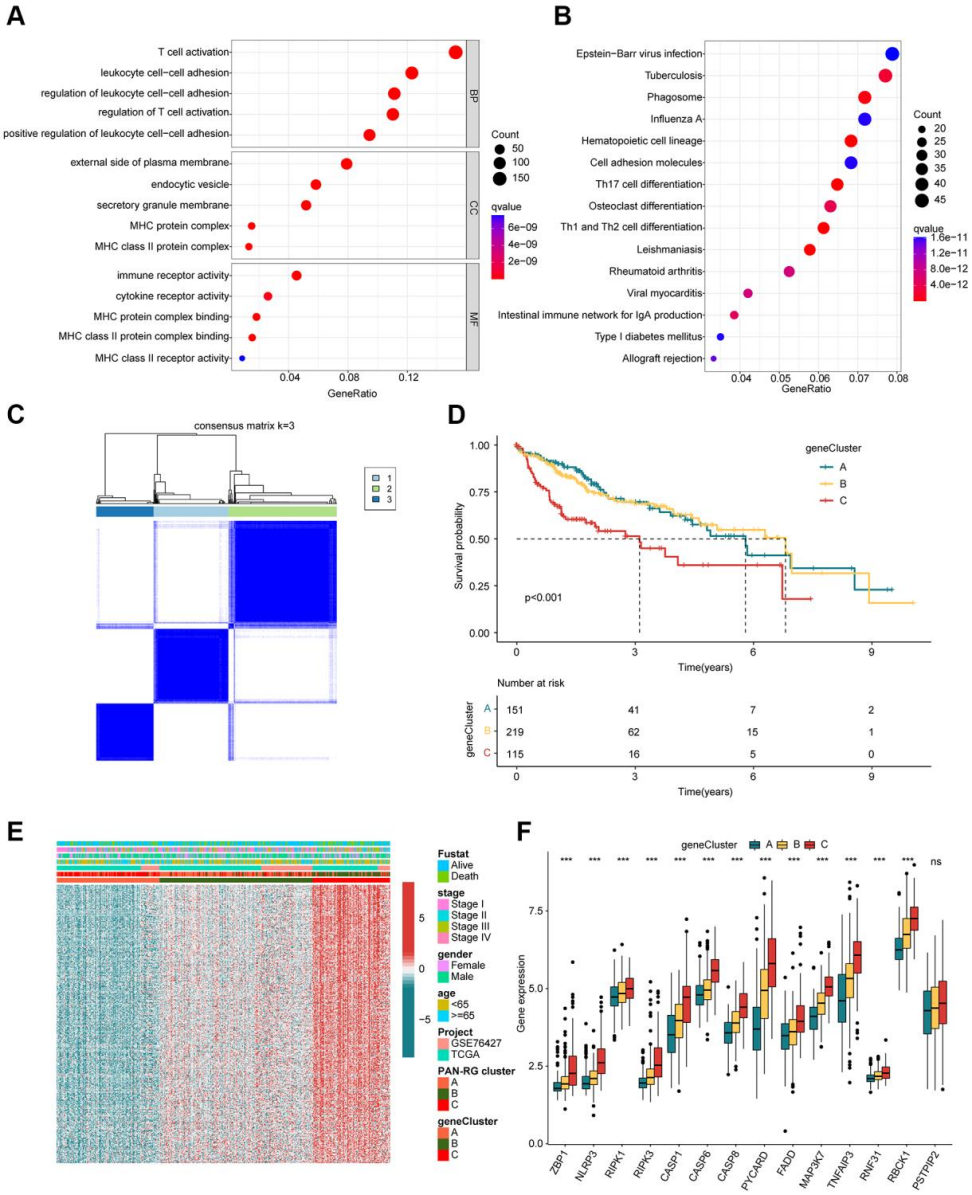
**Identification of DEGs in the PAN-RG molecular subtypes.** (**A**) GO and (**B**) KEGG enrichment assessment of the PAN-RG cluster-based DEGs. (**C**) Unsupervised consensus clustering of the DEGs in HCC. (**D**) Clinical prognosis analysis of HCC in gene-cluster subgroups. (**E**) Heatmap plot illustrates the relationship of clinical variates and prognostic DEGs in PAN-RG- and gene-cluster subgroups. (**F**) PAN-RGs expression landscape of HCC in gene-cluster A, B, and C.

In order to better understand the molecular biological function of the PAN-RG cluster subgroups, differential expression analysis was carried out to identify the DEGs between the PAN-RG cluster subgroups with the screening threshold set at |fold change| ≥ 1 and *p*.adjust < 0.001. On the basis of difference analysis calculation, we obtained 1077 intersection DEGs between the PAN-RG cluster subgroups. GO enrichment analysis exhibited that the PAN-RG cluster-based DEGs were enriched in T cell activation, leukocyte cell-cell adhesion, external side of plasma membrane, and immune receptor activity (Figure 4A). KEGG analysis result displayed that Epstein-Barr virus infection, tuberculosis, and phagosome were enriched of the PAN-RG cluster-based DEGs (Figure 4B). Under the estimation of univariate Cox analysis, we acquired 376 DEGs which associated with clinical prognosis for HCC. On the basis of unsupervised consensus clustering analysis, the HCC samples were successfully divided into 3 cluster subgroups (K = 3), including 151 HCC samples in gene-cluster A, 219 HCC samples in gene-cluster B, and 115 HCC samples in gene-cluster C (Figure 4C). The clinical survival results of HCC samples displayed that the prognosis outcome of HCC in gene-cluster C was even worse than those HCC samples in gene-cluster A and gene-cluster B (*p* < 0.001, Figure 4D). The heatmap plot exhibited the expression profile of prognosis DEGs in the different clinical variates, PAN-RG- and gene-cluster subgroups (Figure 4E). Additionally, we found that the gene-cluster C with worse clinical prognosis displayed higher expression level of PAN-RGs (Figure 4F).

### Establishment of risk subgroups for HCC based on the prognostic DEGs

**Figure 5:**
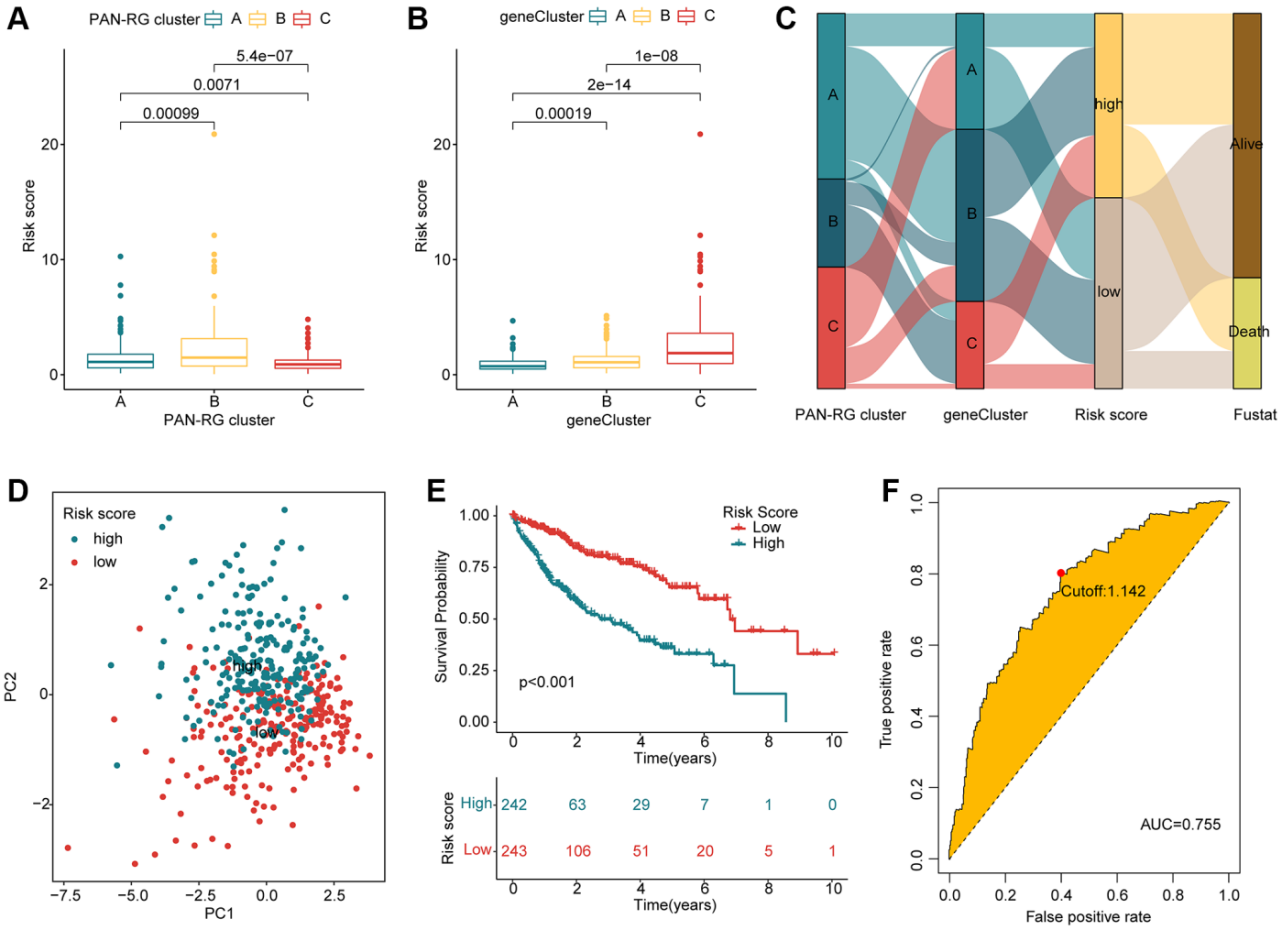
**Evaluation of risk score for HCC samples based on the prognostic DEGs.** (**A**) Risk score of HCC samples in PAN-RGs cluster subgroups. (**B**) Risk score of HCC samples in gene cluster-subgroups. (**C**) Association of risk score and clinical survival status for HCC samples in the PAN-RG- and gene-cluster subgroups. (**D**) The PCA plot of HCC samples in the risk subgroups. (**E**) Prognosis analysis of HCC samples in the risk subgroups. (**F**) The diagnostic effectiveness of the risk score for HCC.

Based on the prognostic factors for HCC, the LASSO algorithm selected 10 feature variates from the 376 DEGs (Supplementary Figure 1). Then, 7 feature variates were obtained for the risk score calculation via multivariate Cox analysis. According to the 7 feature variates, the HCC samples from the TCGA-LIHC and GSE76427 were divided into training cohort and test cohort under the division set at 1:1 via package “caret”. In the PAN-RG cluster subgroups, we observed that the risk score of HCC samples in cluster B was greatly higher than those in the cluster A, and C (Figure 5A). As for gene-cluster subgroups, the HCC samples in gene-cluster C with worse prognosis had remarkable risk score than other gene-cluster subgroups (Figure 5B). The Sankey plot displayed the detail relationship of clinical survival outcome, risk score, PAN-RG cluster, and gene-cluster (Figure 5C). PCA diagram illustrated that the risk score could clearly distinguish the HCC samples with low- and high-risk score (Figure 5D). After the estimation of clinical prognostic outcome for HCC samples, an obvious difference between low- and high-risk groups for HCC was observed in the entire cohort (*p* < 0.001, Figure 5E). In the training cohort and test cohort, the same clinical prognosis outcome was obtained, the HCC samples with low-risk score had better survival rate (Supplementary Figure 2). The ROC of risk score revealed that the AUC was 0.755 (cutoff: 1.142), indicating a favorable diagnostic ability for HCC (Figure 5F). These results demonstrate that establishment of the risk score could divide the HCC samples into different risk subgroups and could accurately evaluate the clinical prognosis for HCC.

### Independent prognostic evaluation of risk score for HCC

**Figure 6:**
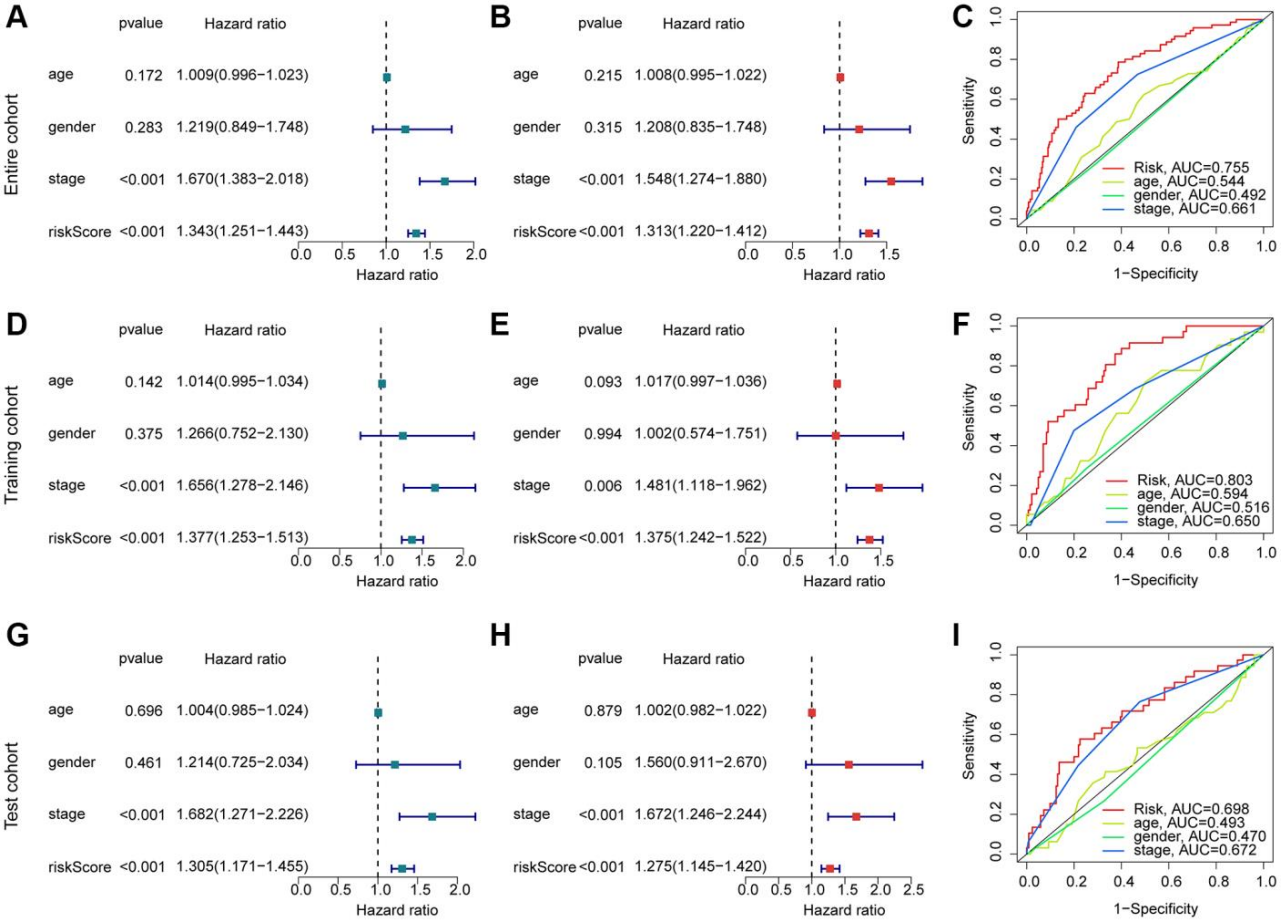
**Exploration of independent prognostic for risk score in HCC.** (**A**, **B**) Univariate and multivariate Cox analysis in entire cohort. (**C**) ROC curve analysis of risk score and clinical variates of HCC. Univariate/multivariate Cox analysis and ROC curve in training cohort (**D**–**F**) and test cohort (**G**–**I**).

Considering the role of the risk score in estimating clinical prognosis outcome for HCC, we further explored the independent prognostic significance of risk score. The clinical variates of HCC were acquired from the TCGA-LIHC and GSE76427, including age, gender and stage. Under the calculation of univariate Cox analysis, the HCC-based stage (HR = 1.670 (1.383–2.018), *P* < 0.001) and risk score (HR = 1.343 (1.251–1.443), *P* < 0.001) were related to poor prognosis for HCC (Figure 6A). For multivariate Cox analysis, the risk score (HR = 1.313 (1.220–1.412), *P* < 0.001) was regarded as an independent prognosis factor for HCC (Figure 6B). The AUC of risk score, age, gender and HCC-based stage was 0.755, 0.544, 0.492 and 0.661, showing a favorable predictive ability of risk score for HCC (Figure 6C). As displayed in Figure 6D–6I, the result of univariate/multivariate Cox analysis suggested that the risk score was an independent prognosis indicator for HCC in the training cohort and test cohort. Moreover, the AUC of risk score in the training cohort and test cohort was 0.803 and 0.698. Taken together, our results illustrate that the risk score is an independent prognostic factor for HCC, showing a favorable diagnostic power than other clinicopathological characteristics.

### Development of nomogram based on risk score and clinicopathological features for HCC

**Figure 7:**
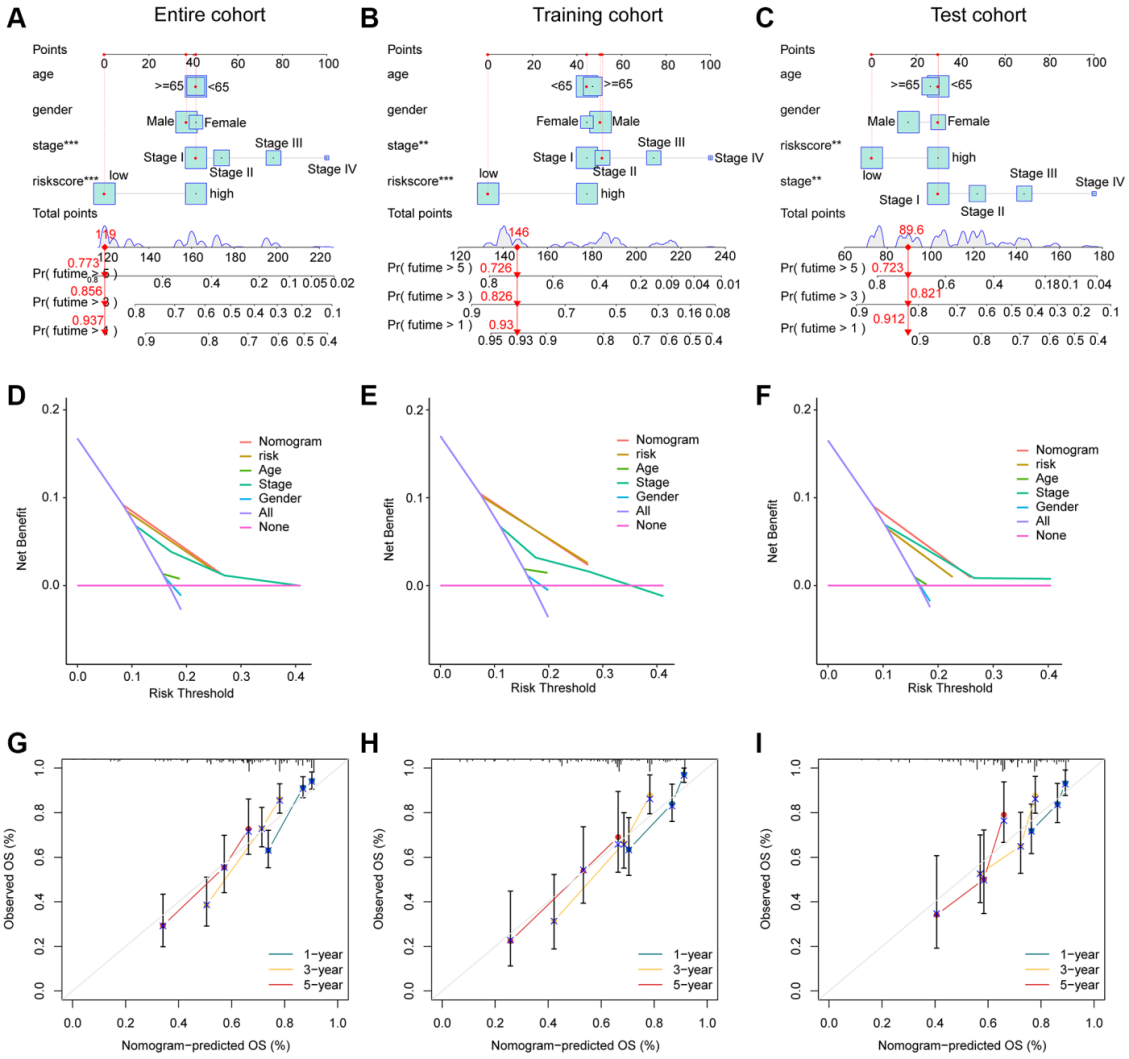
**Development of the nomogram for evaluating the survival probability in HCC.** (**A**–**C**) Nomogram development of risk score and clinical variates in entire, training and test cohorts. (**D**–**F**) DCA curve analysis. (**G**–**I**) Calibration curve estimation between the actual OS and predicted OS.

We developed a nomogram model to explore the one-, three-, and five years clinical outcome for HCC based on the risk score and clinical variates. As illustrated in Figure 7A–7C, the nomogram results in entire, training and test cohorts revealed that the risk score could accurately estimate the survival probability for HCC. The DCA curve exhibited that the development of nomogram in estimating clinical prognosis for HCC was better than other variates in the entire, training and test cohorts (Figure 7D–7F). Furthermore, the calibration analysis showed that the clinical outcome of nomogram assessed in one-, three-, and five years was consisted with the real survival status for HCC, indicating the favorable accuracy of nomogram (Figure 7G–7I).

### The immune infiltration evaluation of risk subgroups for HCC

**Figure 8:**
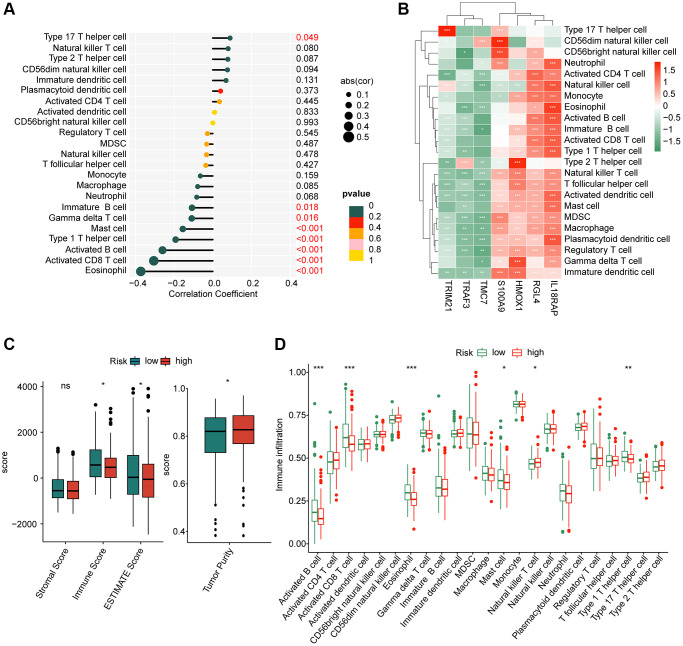
**Assessment of immune infiltration of HCC samples in the risk subgroups.** (**A**) Correlation coefficient of risk score and immune infiltration. (**B**) Correlation analysis of 8 prognostic factors and immune infiltration. (**C**) ESTIMATE score. (**D**) Immune infiltration evaluation of HCC samples in the risk subgroups.

The immune infiltration of HCC samples in the two risk subgroups were further explored in the subsequent investigation. The correlation analysis indicated that the risk score was positively correlated with type 17 T helper cell, but negatively associated with eosinophil, activated CD8^+^ T cell, activated B cell, type 1 T helper cell, mast cell, gamma delta T cell and immature B cell (Figure 8A). Moreover, we explored the potential relationship between 7 independent prognostic factors and immune infiltration, and the heatmap displayed a clearly positive association of 4 prognostic factors (S100A9, HMOX1, RGL4 and IL18RAP) and most of immune cells, however, another 3 prognostic factors (TRIM21, TRAF3 and TMC7) were negatively correlated with most of immune cells (Figure 8B). These results illustrated a potential relation between the risk score and immune infiltration.

Based on the ESTIMATE assessment algorithm, we observed a remarkable difference of immune, ESTIMATE and tumor purity between the two risk subgroups (Figure 8C). Additionally, the ssGSEA algorithm suggested that the proportion of activated B cell, CD8^+^ T cell, eosinophil, mast cell and type 1 T helper cell was higher of HCC samples in the high-risk group, whereas the proportion of natural killer T cell was greatly higher for HCC in the low-risk group (Figure 8D). These results illustrate a clear distinction in immune infiltration of HCC samples in low- and high-risk groups, and associated with the risk score.

### Genetic mutation feature and immunotherapy response of HCC in the risk subgroups

**Figure 9:**
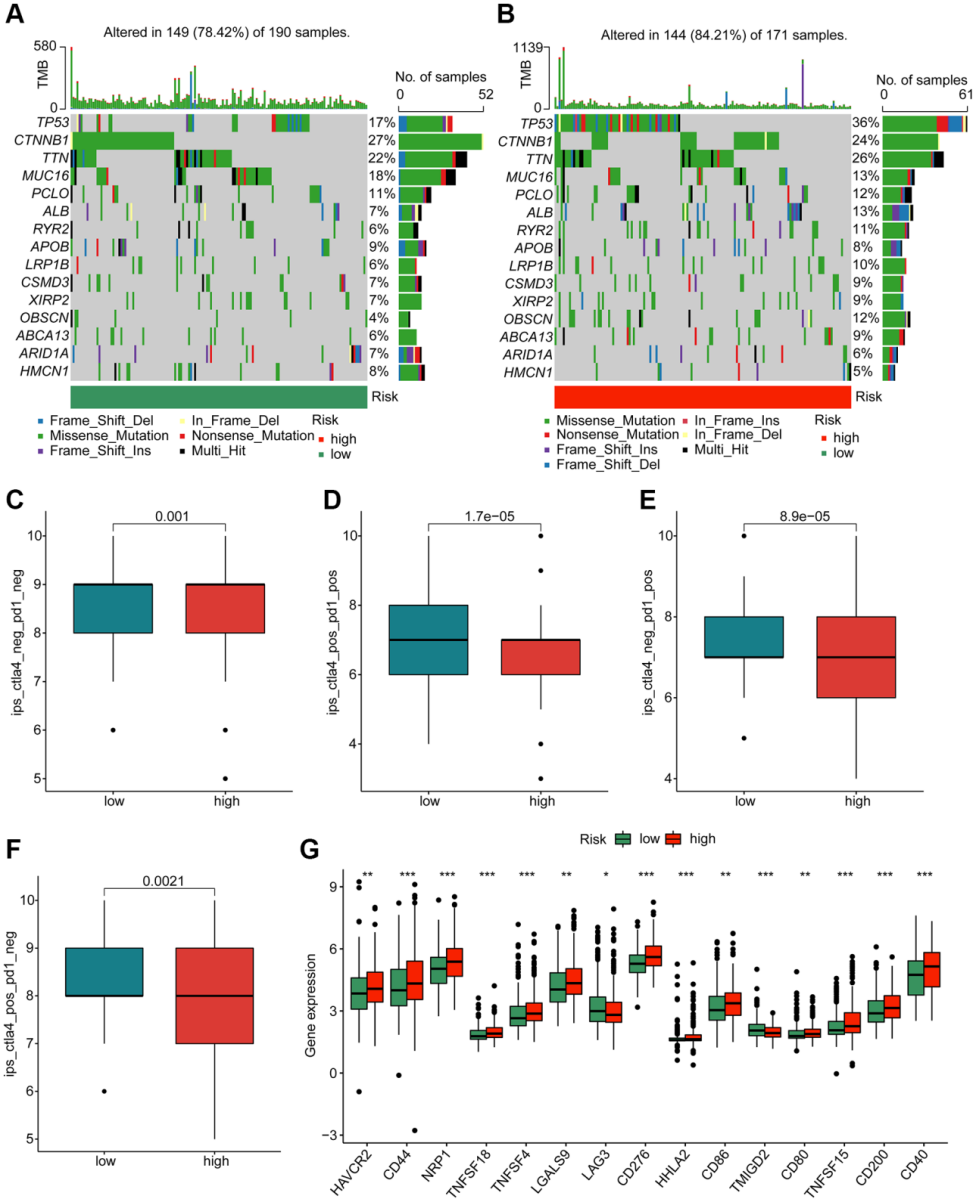
**Genetic mutation characteristic and immunotherapy response of risk subgroups.** Evaluation of somatic mutation landscape of HCC samples in (**A**) low- and (**B**) high-risk groups. (**C**–**F**) Immunotherapy response exploration of risk subgroups. (**G**) Difference analysis of immune checkpoints in the risk subgroups.

In first, we investigated the mutation characteristic of somatic in the risk subgroups for HCC. As illustrated in Figure 9A, 9B, about 149 samples had somatic mutation in 190 samples (78.42%), with lower mutation frequency of TP53 (17%), TTN (22%), ALB (7%) and RYR2 (6%). In high-risk group, we observed 144 samples had somatic mutation in171 samples (84.21%), with lower mutation frequency of CTNNB1 (24%) and MUC16 (13%). Based on the TCIA database, the immunotherapy response to PD-1 and CTLA-4 of HCC samples in the risk subgroups was further explored. The IPS evaluation analysis revealed that the low-risk group was more sensitive to PD-1 and CTLA-4 treatment (Figure 9C–9F). The analysis of immune checkpoints displayed that the expression profile of most immune checkpoints was higher in the high-risk group (Figure 9G). These results demonstrate that the risk score is associated with mutation feature and could indicate the immunotherapy response of HCC samples in the risk subgroups.

### Prediction of potential chemotherapeutic compounds for HCC in risk subgroups

**Figure 10:**
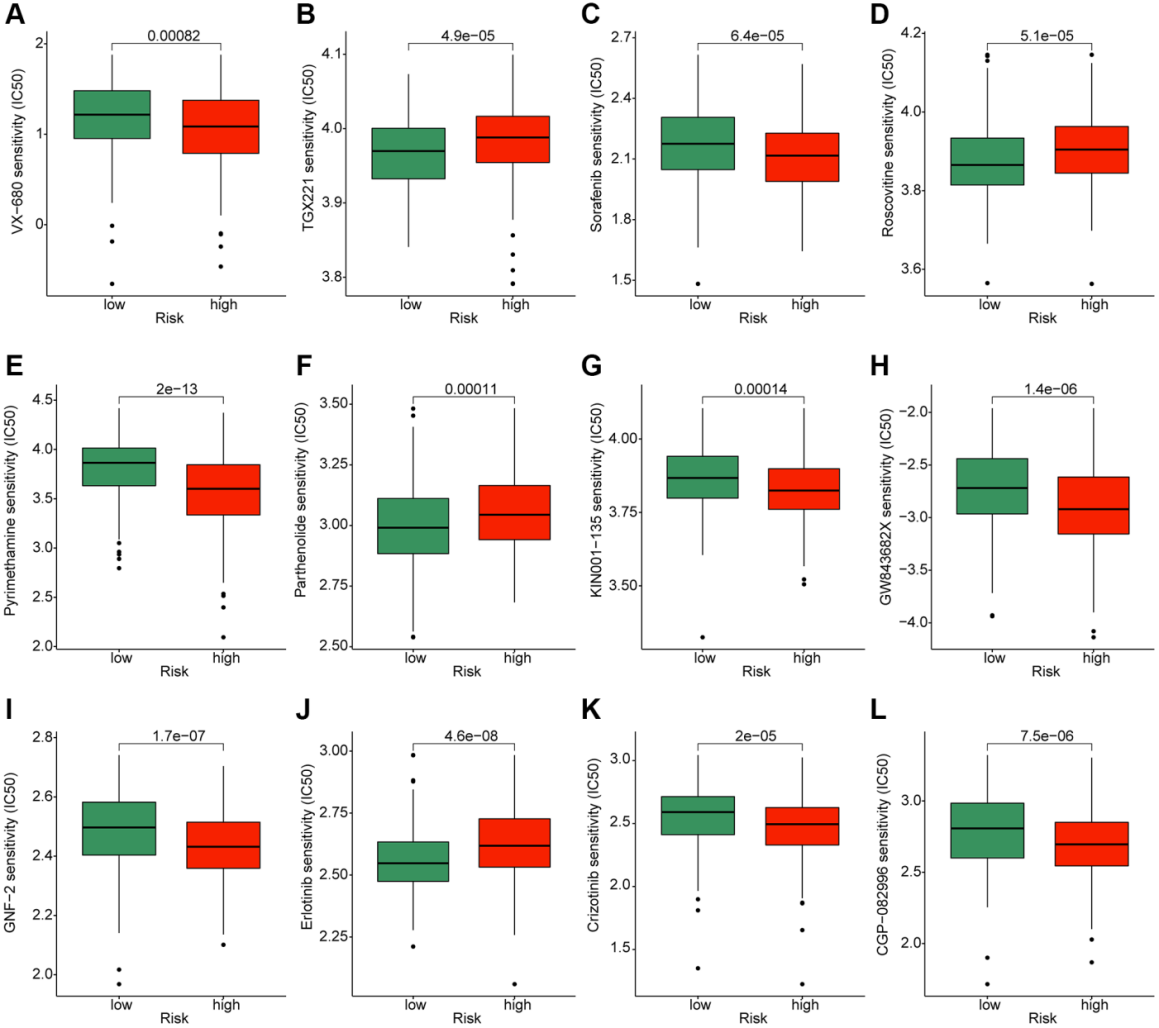
**Exploration of potential chemotherapeutic compounds for HCC in the different risk subgroups.** The drug sensitivity evaluation of (**A**) VX-680; (**B**) TGX221; (**C**) Sorafenib; (**D**) Roscovitine; (**E**) Pyrimethamin; (**F**) Parthenolide; (**G**) KIN001-135; (**H**) GW843682X; (**I**) GNF-2; (**J**) Erlotinib; (**K**) Crizotinib and (**L**) CGP-082996.

To better understand the application of the risk model in the clinical treatment for HCC, we explored several potential chemotherapeutic compounds which may benefit for the treatment of HCC samples in the risk subgroups based on the GDSC database. As implied in Figure 10, the IC50 of VX-680, sorafenib, pyrimethamine, KIN001-135, GW843682X, GNF-2, crizotinib and CGP-082996 in the high-risk group was greatly lower than low-risk group; notably, the IC50 of TGX221, roscovitine, parthenolide and erlotinib was higher in the high-risk group. Overall, these findings illustrate that the risk model could indicate the chemotherapeutic compounds response of HCC samples in the different risk subgroups.

### Analysis of expression profile for prognostic signatures

**Figure 11:**
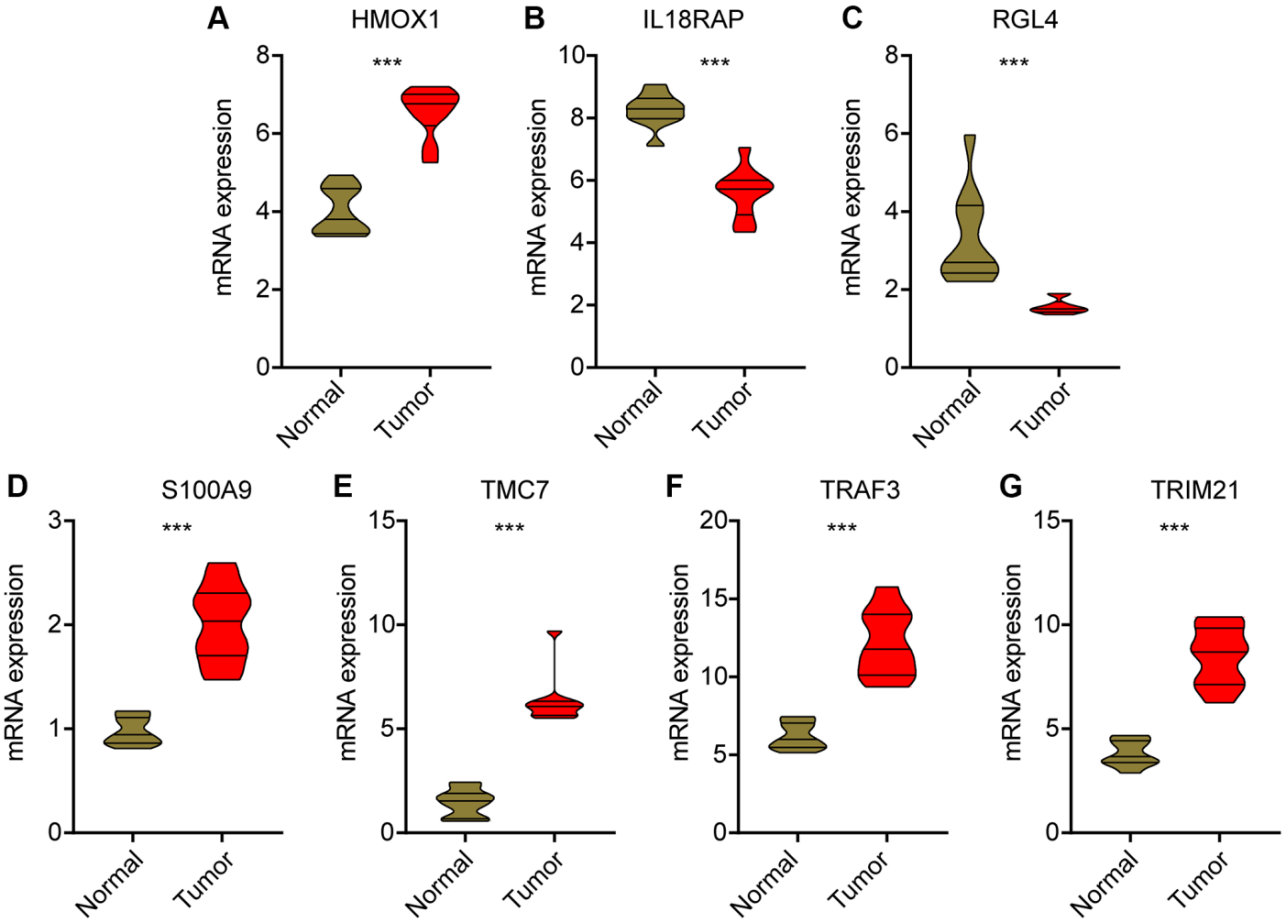
**Validation of mRNA level for prognostic signatures in HCC.** (**A**) HMOX1; (**B**) IL18RAP; (**C**) RGL4; (**D**) S100A9; (**E**) TMC7; (**F**) TRAF3; (**G**) TRIM21.

We used HCC clinical samples to detect the expression levels of selected prognostic signatures. As shown in Figure 11, HCC tumor tissues contain high levels of HMOX1, S100A9, TMC7, TRAF3 and TRIM21, whereas the expression of IL8RAP and RGL4 were higher in the control group (Figure 11A–11G).

## DISCUSSION

In this study, seven PAN-RGs were selected and the prognostic model of HCC was established successfully. Subsequently, through TME analysis, drug resistance analysis, TMB analysis and other studies, we conducted a preliminary discussion on the reasons for the influence of the model on the prognosis.

Among the selected genes, TRAF3 encodes for a protein which plays a role in the regulation of the immune response and is involved in signaling pathways that control cell survival, growth, and differentiation [32]. TRAF3 has been studied in the context of its potential as a therapeutic target in cancer [33]. Some studies have suggested that targeting TRAF3 may be a promising approach for the treatment of certain types of cancer, including HCC [34, 35]. Further research is needed to determine its full implications in the development and progression of HCC. TRIM21 is involved in several processes that play a role in controlling the immune response, including the clearance of viruses and other pathogens and the regulation of antibody production [36]. In addition, some studies have suggested that TRIM21 may play a role in the development and progression of certain diseases, including cancer [37]. The prognostic value of TRIM21 in HCC has been reported [38]. TRIM21 promotes liver cancer by inhibiting the p62-Keap1-Nrf2 antioxidant pathway [39]. In addition, TRIM21 is involved in regulating ACTL6A/MYC axis activity in hepatocellular carcinoma progression [40].

S100A9 is a secreted protein associated with an inflammatory microenvironment, and its expression in tumor tissues is associated with poor survival in HCC patients [41]. Reported mechanisms include that S100A9 enhances cancer stem-like properties of hepatocellular carcinoma [42]. Activation of RAGE/TLR4-ROS signaling pathway leads to the formation of Neutrophil extracellular traps (NETs) that promote HCC growth and metastasis [43]. Depletion or pharmacological inhibition of S100A9 significantly inhibited HCC growth and metastasis ability [44]. Thus, targeting S100A9 may be a promising therapeutic strategy for patients with hepatocellular carcinoma. HMOX1 has been shown to play a role in the regulation of oxidative stress and inflammation. In HCC, HMOX1 can reduce the sensitivity of hepatocellular cancer cells to sorafenib, which may be mediated by regulating the expression of ABC transporters [45, 46]. At the same time, as an important target of NRF2 and KEAP1, HMOX1 may be involved in ferroptosis induction [47]. The exact role of IL18RAP, RGL4 and TMC7 in cancer and its potential as a therapeutic target or marker is still not well understood, and further research is needed to determine its full implications in the development and progression of cancer.

We provide the first evidence that PANoptosis plays a role in HCC. In the PANoptosis process, as an important driving protein, caspase-8 plays an active but complex role. Activated caspase-8 promotes the activation of caspase 3 in the process of inducing apoptosis, leading to secondary necroptosis or pyroptosis, and thus inhibiting tumor growth [48]. However, the opposite effect of caspase-8 in HCC has also been reported. It promotes tumor cell migration and inhibits Fas induced apoptosis by assembling caspase-8/RIPK1/FADD/cFLIP complex [49]. In addition, other reports have suggested the role of PANoptosis in the evaluation of HCC treatment. The antitumor effect of oxaliplatin is achieved at least in part by increasing the level of intracellular reactive oxygen species triggering PANoptosis [16]. Oxaliplatin can be used for hepatic arterial infusion chemotherapy [50] due to its unique pharmacokinetic, cytotoxic and immunological properties. Thus, the level of PANoptosis is theoretically helpful in evaluating the selection of drugs during hepatic arterial perfusion chemotherapy. In addition, IFN treatment can be affected by PANoptosis [19]. IFN-γ is approved as a cytokine for several cancer species and has been shown to induce apoptosis of HCC cells [51]. IFN was also used for chronic hepatitis B [52]. As a critical risk factor for HCC, whether the therapeutic effect of hepatitis B virus infection is affected by PANoptosis level needs to be further demonstrated.

Additionally, 5 PAN-RGs were shown to be prognostic risk factors (MAP3K7, CASP8, RBCK1, FADD, CASP6). Caspase (CASP) is a conserved family member involved in signaling and execution of apoptotic pathways [53]. Its members CASP6 and CASP8 have been shown to be associated with HCC prognosis, mediated by the connexin32 (Cx32)-Src axis, and the inactivation of caspase-8 contributes to the activation of necrotic apoptosis in HCC cells [54–56]. MAP3K7 has been reported to affect mTOR phosphorylation and expression levels, and may contribute to HCC tumorigenesis via the MAP3K7-mtor axis [57]. RIPK1 kinase activity can induce FADD-dependent apoptosis. In HCC, FADD and RIPK1 act synergistically to influence tumorigenesis through mediating apoptosis [26]. These PAN-RGs provide interesting clues to the mechanism by which PANoptosis affects prognosis.

The role of eosinophils in liver hepatocellular carcinoma (HCC) prognosis is still not well understood and the subject of ongoing research. Some studies have found that high numbers of eosinophils in the tumor microenvironment (TME) are associated with HCC prognosis [58]. Our results showed lower levels of eosinophils in the high-risk group, indicating the association between eosinophils and a better prognosis in HCC. Eosinophil has shown its anti-tumor effects in HCC [59]. Additionally, increased percentage of eosinophils may also reflect tumor cell death and indicate the responding to treatment or other procedures [60]. Lower blood eosinophil counts were reported to be associated with poor therapeutic responsiveness in patients with hepatocellular carcinoma treated with sorafenib [61]. One hypothesis is that eosinophils contribute to tumor angiogenesis, inflammation, and immunosuppression in the TME, which can impact the growth and spread of the tumor [62, 63]. However, the exact mechanism by which eosinophils impact HCC prognosis is not yet clear, and further research is needed to determine their potential as targets for new treatments.

To summary, we established a prognostic model consisting of seven PAN-RGs, and verified the significance of prognostic prediction and the possible clinical value of selected targets through public databases and our own clinical specimens. However, this article only provides a preliminary idea of the principles behind this risk stratification. Further research on this basis will provide new ideas and targets for HCC treatment.

## Supplementary Materials

Supplementary Figures

Supplementary Tables
