# Supplementary material for: Characteristic of molecular subtypes based on PANoptosis-related genes and experimental verification of hepatocellular carcinoma
Source: Aging (Albany NY). 2023 May 12;15(10):4159–81. doi: 10.18632/aging.204720 (PMC10258029; doi:10.18632/aging.204720)
Supplement: Supplementary Figures [file aging-15-204720-s001.pdf]

SUPPLEMENTARY FIGURES

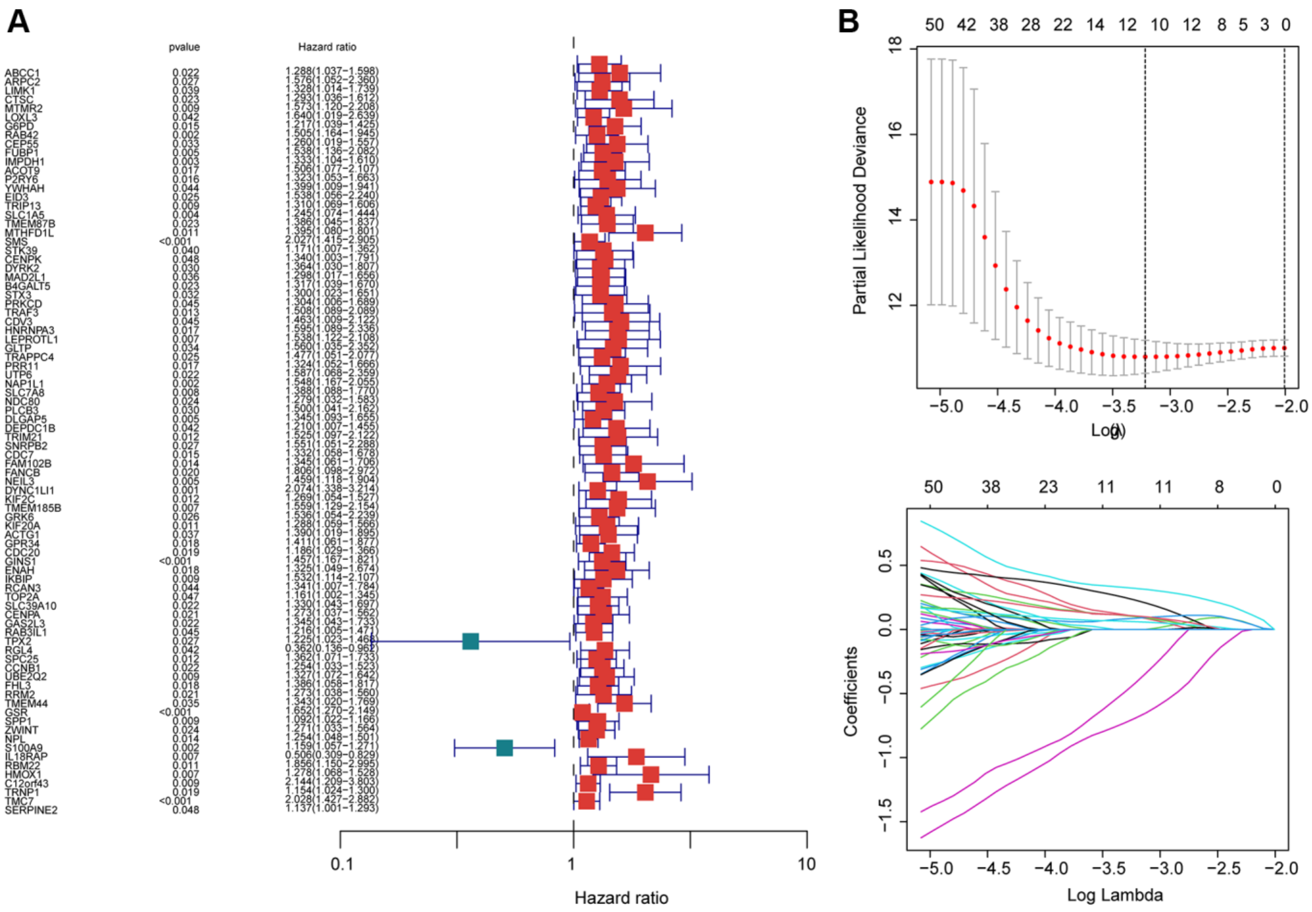

Supplementary Figure 1. The LASSO-univariate Cox algorithm of 376 DEGs. (**A**) Univariate Cox analysis of DEGs. (**B**) Identification of feature prognostic DEGs based on LASSO model.

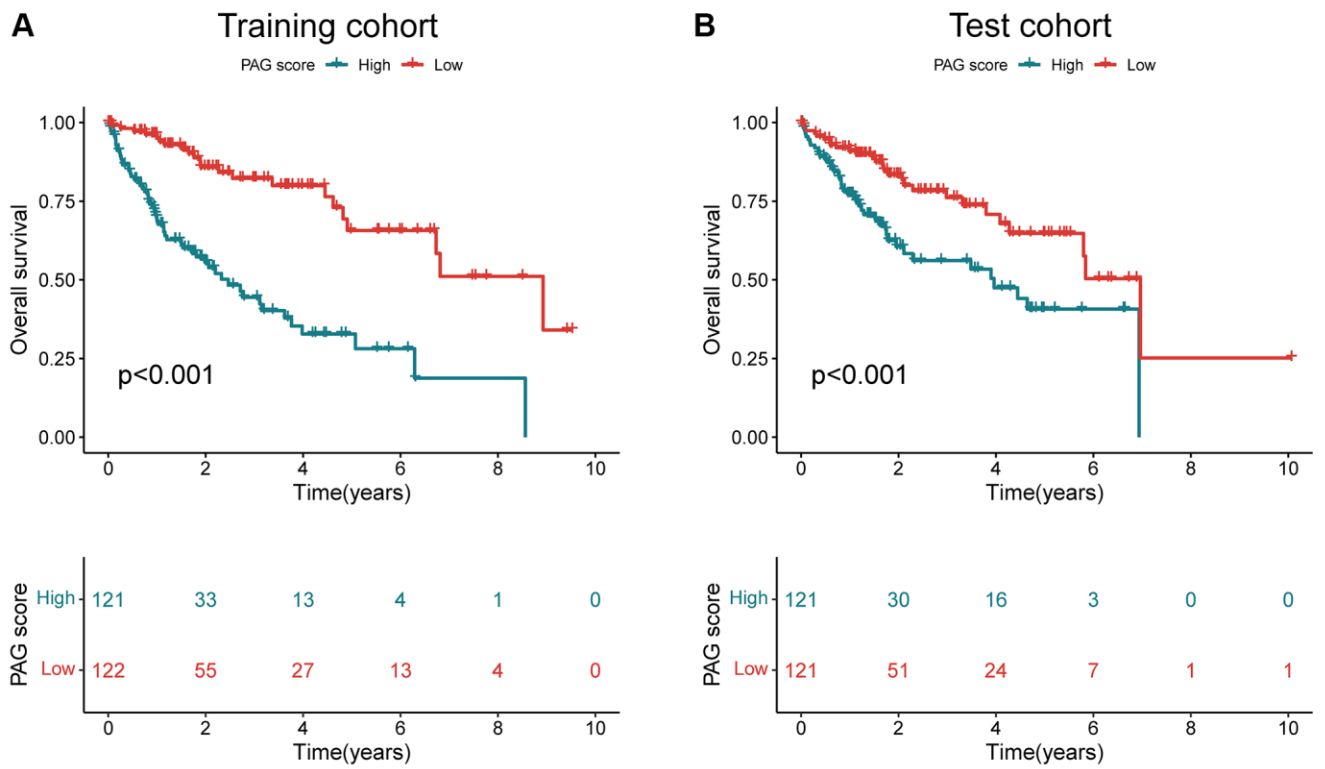

Supplementary Figure 2. Development of risk model based on the PAG score in the training and test cohorts. The clinical prognostic analysis of HCC samples with low- and high PAG score in the (**A**) training and (**B**) Test cohorts.
