# Supplementary material for: Characteristic of molecular subtypes based on PANoptosis-related genes and experimental verification of hepatocellular carcinoma
Source: Aging (Albany NY). 2023 May 12;15(10):4159–81. doi: 10.18632/aging.204720 (PMC10258029; doi:10.18632/aging.204720)
Supplement: Supplementary Tables [file aging-15-204720-s002.pdf]

SUPPLEMENTARY TABLES

Supplementary Table 1. The gene list of PANoptosis-related genes.

Gene symbol
ZBP1
NLRP3
RIPK1
RIPK3
CASP1
CASP6
CASP8
PYCARD
FADD
MAP3K7
TNFAIP3
RNF31
RBCK1
PSTPIP2

Supplementary Table 2. Gene specific primer pairs.

RGL4	F	CCCCCAGAGAGTCCAGATGA
	R	TTTCCTGCAGAACTCGGACC
IL18RAP	F	AGAGCACTTCCTACTGAAAGAGG
	R	GGCTACACCTTCAGCTGTCT
S100A9	F	CGGCTTTGACAGAGTGCAAG
	R	GCCCCAGCTTCACAGAGTAT
HMOX1	F	TCCTGGCTCAGCCTCAAATG
	R	CACGCATGGCTCAAAAACCA
TMC7	F	GCGTCCTCATCCAATCCAGT
	R	GTCTCGGTCAACAGCTGGAA
TRAF3	F	ACCGCGAGAACTCCTCTTTC
	R	TCAGGGACAAAAACTGGCGT
TRIM21	F	CCCCTCTAACCCTCTGTCCA
	R	CTGCTAAAGCTCGCTTGCTG
